# Monitoring Volatile Organic Compounds in Different Pear Cultivars during Storage Using HS-SPME with GC-MS

**DOI:** 10.3390/foods11233778

**Published:** 2022-11-23

**Authors:** Guanwei Gao, Xinnan Zhang, Zhen Yan, Yang Cheng, Haifei Li, Guofeng Xu

**Affiliations:** 1 Ministry of Agriculture and Rural Affairs, Institute of Pomology, Chinese Academy of Agricultural Sciences, Xingcheng 125100, China; 2 Laboratory of Quality & Safety Risk Assessment for Fruit, Xingcheng 125100, China; 3 Key Laboratory of Germplasm Resources Utilization of Horticultural Crops, Ministry of Agriculture and Rural Afffairs, Xingcheng 125100, China

**Keywords:** volatile organic compounds, pear, cultivar, storage, HS-SPME, GC-MS

## Abstract

Aroma, which plays an essential role in food perception and acceptability, depends on various mixture of volatile organic compounds (VOCs). Meanwhile, as a field of metabolomics, VOC analysis is highly important for aroma improvement and discrimination purposes. In this work, VOCs in pear fruits were determined via headspace solid-phase micro-extraction (HS-SPME) combined with gas chromatography–mass spectrometry (GC–MS) to study variations among different cultivars and storage stages. In 12 cultivars of pear fruits, a total of 121 VOCs were quantified, including 40 esters, 32 alcohols, 16 aldehydes, 13 alkenes, 11 ketones, 4 acids, and 5 other compounds. The types and amounts of VOCs in different cultivars varied dramatically, which were in the range of 13–71 and 3.63–55.65 mg/kg FW (fresh weight), respectively. The Dr. Guyot cultivar showed the highest level of VOCs, both in type and amount. After 21 days storage at 4 °C, total concentration of VOCs increased from initial levels of 50.76 to 101.33 mg/kg FW. Storage at 20 °C made a larger contribution to production for VOCs than that at 4 °C, resulting in the maximum content of VOCs (117.96 mg/kg FW) in fruit after 14 days storage at 4 °C plus 7 days at 20 °C. During storage, the content of esters showed a gradual increase, while the content of alcohols and aldehydes decreased. Based on the results presented, related alcohols were recognized as the intermediates of conversion from aldehydes to esters.

## 1. Introduction

Aroma is considered an important characteristic for food quality and plays an essential role in food perception and acceptability [1]. Each food has a distinctive aroma, which depends on various mixtures of volatile organic compounds (VOCs) [2,3]. However, the content of desired VOCs in raw food material is often low, making production of natural flavor costly [4]. Economic value and large-scale production of highly prized foods have made them an easy target for adulteration and fraud [5]. This phenomenon has resulted in adverse economic and human health effects [6]. Therefore, suitable analytical approaches are urgent needs for food authentication [5]. As a field of metabolomics, VOC analysis is highly important for tracing and authentication of food [7]. The first step is to gather VOC information and then search a number of biomarkers.

Pear (*Pyrus*) is one of the most economically important temperate fruits around the world for its nutritional and sensory properties [8]. Based on the FAO Statistical Database, annual worldwide production of pear fruit was nearly 40 million tons in 2020 [9]. Environmental circumstances, genetic attributes, and storage conditions are largely responsible for production of VOCs. Several investigations have focused on the influence of VOCs in various pear cultivars [10,11,12,13], storage conditions [14,15], and post-harvest treatments [16,17]. Pear species around the world include two separate groups, namely, occidental and oriental pears. Furthermore, at least 22 recognized species with over 5000 accessions are included in the genus *Pyrus*. Therefore, information about VOCs is the foundation for a breeding program of pear cultivars aimed at improving their aroma.

Gas chromatography–mass spectrometry (GC–MS) combined with headspace solid-phase micro-extraction (HS-SPME) is a simple, quick, and cheap technique without organic reagent requirements [18]. It has been widely applied in determination of VOCs in apple [19,20], orange [21,22], and other fruits [23]. In the present study, the composition and profiles of VOCs in 12 cultivars of pear fruits were analyzed. After that, changes of VOCs in pear fruit during storage were investigated to analyze the conversion pathway of flavor compounds. Specific VOCs for each cultivar could be used as potential markers for discrimination purposes. In addition, results from this work could provide preliminary data about VOCs in pear cultivars aimed at improving fruit aroma quality.

## 2. Materials and Methods

### 2.1. Reagents and Chemicals

Cyclohexanone (>99%) used as internal standard was obtained from Tianjin chemical reagent Ltd. (Tianjin, China). Standard solution was prepared with 10% methyl alcohol. Methyl alcohol was HPLC grade and was supplied by Fisher Scientific (Pittsburgh, PA, USA). Purified water was purchased from Wahaha Foods Co., Ltd. (Hangzhou, China). Sodium chloride (NaCl) used in all the experiments was provided by Agela Technologies (Newark, DE, USA).

### 2.2. Sample Collection

Pear samples with suitable maturity were purchased from local markets in different provinces of China. The origin, group, scientific name, province, geographic point, sampling time, and characteristic taste are listed in Table 1. In addition, photographs of pear fruits are displayed in Figure S1. Each sample was collected in triplicate. All samples were packed and delivered immediately to the laboratory at Xingcheng China, and then immediately analyzed for composition and concentration of VOCs.

Results of VOC analysis in 12 pear cultivars showed that Dr. Guyot pear contained a high level of VOCs. Therefore, Dr. Guyot was selected for VOC analysis during storage. About 100 Dr. Guyot pear fruits were stored under low temperature (4 °C) immediately after receiving them. After 14 days of storage at 4 °C, the pear fruits were randomly divided into two groups, one of which was further stored for 7 days at 4 °C. Another group was removed from storage at 4 °C and held for 7 days at 20 °C. Dr. Guyot pear fruits before and after 14, 21 days storage at 4 °C and 14 days storage at 4 °C plus 7 days at 20 °C the groups were sampled (ten fruits each). Pears stored at 4 °C were kept at room temperature for 2 h before sample preparation.

### 2.3. Sample Preparation

Sample preparation was performed at room temperature (about 20 °C). The fruit core of each pear was removed and discarded. Then, the fruit was sliced using a sharp stainless steel knife. Before homogenization with a commercial juice extractor, the sliced pear was mixed with NaCl (1:1, m/m). After that, the homogenized mixture (10.0 g) and 0.1 mL standard solution of cyclohexanone at 0.2 mg/mL were transferred into a SPME vial. Before sealing, purified water was added into the vial to reach a total of 10 mL volume. All the samples were stored at −20 °C until analysis.

### 2.4. HS-SPME Conditions

VOCs were extracted using a divinylbenzene/carboxen/polydimethylsiloxane (DVB/CAR/PDMS) SPME fiber (50/30 µm thickness), which was purchased from Sigma-Aldrich (Supelco, Bellefonte, PA, USA). Extraction of VOCs from samples was achieved with the AOC 6000 auto-sampler (Shimadazu, Tokyo, Japan). Before each analysis, fiber was conditioned at 250 °C for 10 min. For enhancing the dissociation of VOCs, samples were incubated at 80 °C for 15 min. SPME fiber was then inserted into the headspace of the vial for 15 min (agitator speed was 300 rpm) to extract VOCs at 80 °C.

### 2.5. GC-MS Analysis

Samples were analyzed using a Shimadazu 2010plus GC equipped with a QP2010 mass spectrometry system (Shimadazu, Tokyo, Japan). VOCs on fiber were desorbed at 200 °C for 1 min at the injector port using the splitless mode. The chromatograph was fitted with a HP-INNOWAX capillary column (60 m × 0.25 mm × 0.25 μm, Agilent Technologies, Santa Clara, CA, USA). Initial column temperature was adjusted at 50 °C for 1 min, followed by an increase of 2 °C/min to 180 °C (1 min holding time), and finally an increase at 10 °C/min to 230 °C, held for 10 min. Helium (99.999% purity) was used as the carrier gas with a constant flow of 1.0 mL/min.

MS analysis was performed under electron ionization (EI) mode with an ionizing energy at 70 eV. The ion source temperature in the detector was 230 °C. Mass spectra were scanned in the range of *m/z* 50–500. Identification of target compounds was performed using spectral similarity with the NIST 17s library database (matching quality higher than 90%). In addition, the matching quality of each peak of the VOCs was checked.

### 2.6. Data Processing and Statistical Analysis

Quantification for VOCs was performed by the internal standard method, in which the content of each compound was normalized to that of cyclohexanone. Peak area was measured by integration and was used for quantitative analysis. The following formula was used for VOC quantification:

$$C_{v}=\frac{\frac{A_{v}}{A_{i}}\times C_{i}\times V_{i}}{m\times 10^{-3}}$$

*C_v_*: the content of VOCs (mg⁄kg FW); *A_v_*: peak area of VOCs; *A_i_*: peak area of internal standard; *C_i_*: the concentration of internal standard (0.2 mg⁄mL); *V_i_*: the volume of internal standard (0.1 mL); *m*: mass of sample (5.0 g).

All data were generated in triplicate experiments and contents of VOCs were reported as means ± standard deviations. Excel 2016 software was used for data analysis and graphic presentation. Principal component analysis (PCA) was used to detect clustering and to establish relationships between cultivars and VOCs using SIMCA14.1 (MathWorks, Natick, MA, USA).

## 3. Results and Discussion

### 3.1. Identification and Quantification of VOCs

Salt addition was used in a previous study to enhance the dissociation of VOCs [18]. However, browning of pear fruit occurred rapidly and resulted in color changes (Figure S2b) and loss of flavor. In accordance with our previous study, NaCl was mixed with sliced pear (1:1, m/m) before homogenization to prevent browning [20]. As can be seen in Figure S2a, the color of the pear matrix was less intense and prevented browning from developing.

**Figure 1:**
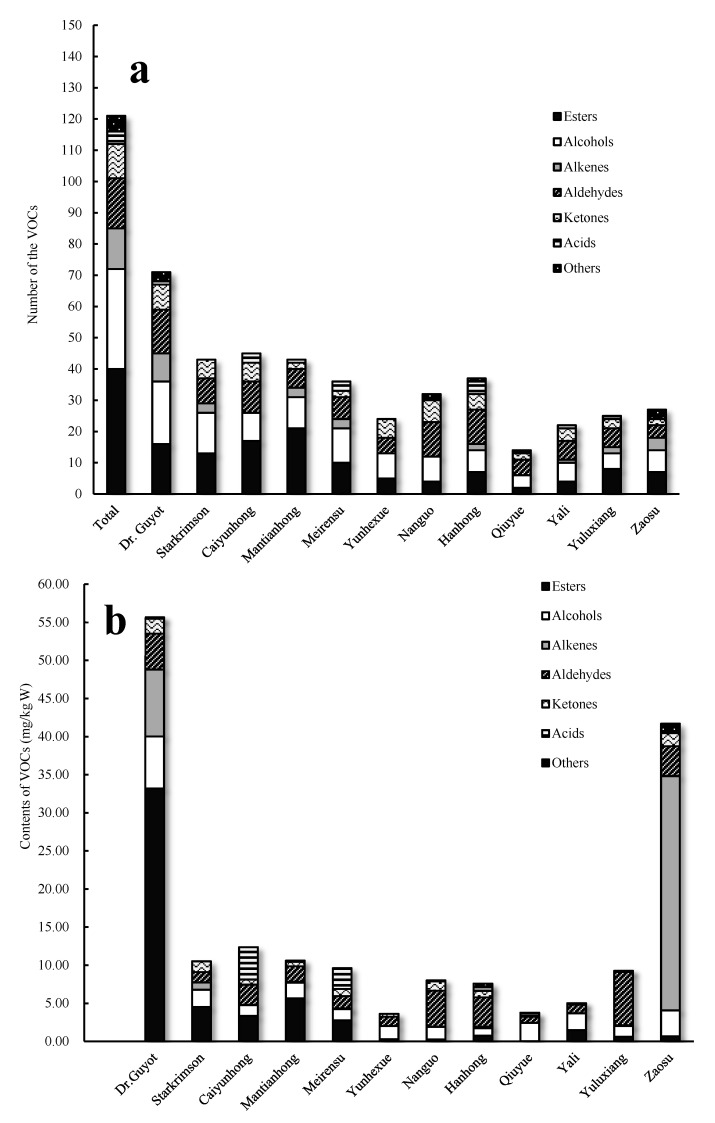
The number (**a**) and contents (**b**) of VOCs detected in 12 cultivars of pear fruits. Note: *n* = 3, equivalent of cyclohexanone.

**Figure 2:**
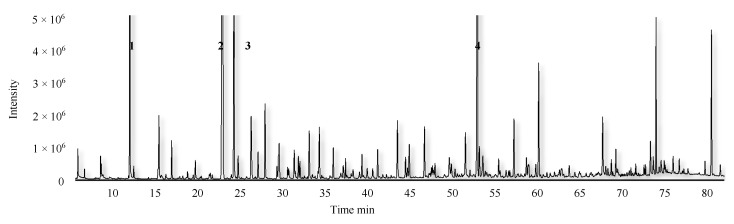
Total ion chromatograms of GC-MS in Dr. Guyot pear. Note: 1, butyl acetate; 2, hexyl acetate; 3, cyclohexanone (internal standard); 4, (E,E)-α-farnesene.

In 12 cultivars of pear fruits, a total of 121 VOCs were identified and quantified, including 40 esters, 32 alcohols, 16 aldehydes, 13 alkenes, 11 ketones, 4 acids, and 5 other compounds. The number of VOCs in different pear cultivars varied dramatically and ranged from 13 to 71 (Figure 1a). It was noteworthy that Dr. Guyot pear had the highest number of VOCs (Figure 2).

As shown in Figure 1a, between 32 and 45 different VOCs were detected in Starkrimson, Caiyunhong, Mantianhong, Hanhong, Meirensu, and Nanguo pears, while less than 30 VOCs were found in Zaosu, Yuluxiang, Yunhexue, Yali, and Qiuyue pears. Among them, Hanhong, Qiuyue, Yali, Yuluxiang, and Zaosu are white pears (Table 1). In this study, Dr. Guyot (71) and Starkrimson (43) pears produced considerably more types of VOCs than did white pears (13–37).

Significant differences in amounts of VOCs among pear cultivars were also observed (Figure 1b). Dr. Guyot pear had the highest total content of VOCs (55.65 mg/kg FW, fresh weight), followed by Zaosu (41.77 mg/kg FW). Meanwhile, VOC content in Starkrimson, Mantianhong, and Caiyunhong pears were 10.51 mg/kg FW, 10.64 mg/kg FW, and 12.36 mg/kg FW, respectively. In contrast, the other seven pear cultivars produced a low level of VOCs, with a content of less than 10 mg/kg FW.

Aroma is the mixture of VOCs with various compositions and concentrations [24]. In this study, esters, alcohols, and aldehydes were the main groups of VOCs (Figure 1), which were consistent with the findings obtained in occidental pears [11], and Chinese white pears [13]. However, Zaosu pear was characterized as having a high content of alkenes (Figure 1b), a similar characteristic to that found in Fuji apple [20]. It was interesting to note that one of the maternal parents of Zaosu pear is named ‘apple-pear’ in China.

Esters. With fruity notes, esters are the most significant contributors to pear aroma. According to a previous study, esters are the dominant VOCs in pear fruit, both in type and amount [11]. In this study, similar results were also observed, especially in occidental, and sand pears (Figure 1).

As the predominant ester, acetyl esters were commonly identified in pears (Table S1). Among them, butyl acetate and hexyl acetate were the main esters [25], and were remarkable concentrated in Dr. Guyot pear (at 7.18 mg/kg FW and 19.91 mg/kg FW, respectively). Methyl palmitate was detected in all pears, with the exception of Meirensu. In addition, heptyl acetate, ethyl oleate, and ethyl palmitate were detected only in Dr. Guyot pear, with concentrations over 0.10 mg/kg FW (Table S1).

Alcohols. Next to esters, alcohols constituted the second-largest group relative to the amount of VOCs in all pear cultivars [11]. In this study, the number of alcohols was highest in Dr. Guyot (20), and lowest in Qiuyue (4) pears. Concentrations of these compounds ranged from 0.99 to 6.79 mg/kg FW. In Yunhexue and Yali pears, alcohols comprised the largest group, both in type and amount.

In addition to the commonly detected compounds, some alcohols were detected specifically in one or two occidental pear cultivars [11]. Similarly, 19 alcohols were detected only in one or two cultivars, some of which had a high concentration (Table S1). For example, bergamotol and 1-butanol were detected only in Dr. Guyot pear with concentrations of 1.45 mg/kg FW and 0.84 mg/kg FW, respectively. The compound 5-decen-1-ol was detected in Mantianhong (0.58 mg/kg FW), while it was absent in the other pear cultivars. In addition, 1-hexanol and 3,5-di-tert-butylphenol were detected in all cultivars with contents of 0.05–3.12 mg/kg FW and 0.02–0.60 mg/kg FW, respectively.

Aldehydes. Aldehydes produce a wide range of special flavors and odors, even at trace amounts [26]. These compounds are predominant in the immature fruit, but some aldehydes become imperceptible after ripening [27]. In this study, the number of aldehydes detected in pears was 4–14, while the contents were 0.82–6.96 mg/kg FW.

Volatile C6 and C9 aldehydes are important contributors to green, grassy characteristic flavors in pear [11]. Among them, hexanal and 2-hexanal were found in all pears at concentrations of 0.10–4.60 mg/kg FW (Table S1). The compound 2-octenal, with the content of 0.03–0.89 mg/kg FW, was also detectable in all cultivars. Nonanal (C9) provided a strong smell of grease and sweet orange flavor. It is commonly detected in apples [19] and pears [13]. In the present work, nonanal was absent only in Yunhexue pear, and its content was 0.12–0.84 mg/kg FW in the other cultivars.

Ketones. Ketones are the main fruity, sweet flavor compounds in fruit. However, they are undetectable in 13 among 33 *Pyrus ussuriensis* cultivars [13]. In this study, ketones were present in all 12 cultivars, with a total number of 11. There were 8 ketones found in Dr. Guyot pear. Concentration of ketones was highest in Dr. Guyot (1.96 mg/kg FW), followed by Zaosu (1.72 mg/kg FW), Starkrimson (1.41 mg/kg FW), and Nanguo (1.25 mg/kg FW), whereas the lowest was in Yali pear (0.11 mg/kg FW). With the exception of Qiuyue, damascenone was present in all cultivars, with concentrations ranging from 0.04 to 1.50 mg/kg FW.

Alkenes. There were 13 alkenes detected in 12 pear cultivars, 9 of which were found in Dr. Guyot pear (Table S1). It should be noted that, in Zaosu pear, the most abundant volatile was (E,E)-α-farnesene (29.51 mg/kg FW), giving this pear a characteristically high content of alkenes. In addition, (E,E)-α-farnesene had a high content in Dr. Guyot pear (7.69 mg/kg FW), which was next only to the content of hexyl acetate, and butyl acetate.

Acids. A total of four acids were present in all pears, including hexanoic, octanoic, nonanoic, and decanoic. All were found in Hanhong pear. Meanwhile, all were absent in Starkrimson, Yunhexue, Nanguo, and Zaosu pears. Nonanoic acid was the most abundant acid compound, with high concentration in Caiyunhong (3.81 mg/kg FW) and Meirensu (2.31 mg/kg FW) pears.

### 3.2. Principal Component Analysis of VOCs

PCA analysis was used to extract important information from VOCs found in 12 pear cultivars. The scores scatter plot of 12 pear cultivars and the corresponding loadings plot of VOCs are shown in Figure S3. The 12 pear cultivars could be divided into three groups on the basis of the relationships between cultivars (scores) and their VOCs (loadings). The first group included one cultivar, Dr. Guyot, which contained the most types and amounts of VOCs. The second group contained three sand pear cultivars (Caiyunhong, Mantianhong, and Meirensu), which were characterized by high relative contents of ethyl butyrate (1), ethyl hexanoate (9), ethyl tiglate (10), and ethyl octanoate (18). The third group was composed of the other eight cultivars, which contained high relative levels of 1-hexanol (47), 3-hexen-1-ol (48), and hexanal (73). Among cultivars in the third group, Hanhong, Qiuyue, Yali, Yuluxiang, and Zaosu are Chinese white pears, Starkrimson is an occidental pear, Nanguo belongs to the Akiko pear group, and Yunhexue is a cross between a sand and a white pear.

### 3.3. Changes in VOCs during Storage

According to the results presented above, Dr. Guyot pear fruit contained the most types and amounts of VOCs among 12 cultivars. Therefore, Dr. Guyot pear was selected for further VOC analysis during storage. Table 2 shows the composition of VOCs with concentration of at least 0.10 mg/kg FW. After 14- and 21-days storage at 4 °C, total concentration of VOCs increased from an initial concentration of 50.76 to 71.38 and 101.33 mg/kg FW, respectively. Additionally, storage at 20 °C contributed to a larger production of VOCs than storage at 4 °C. Thus, the maximum content of VOCs (117.96 mg/kg FW) appeared in fruit after 14 days storage at 4 °C plus 7 days at 20 °C.

According to previous studies, esters play an essential role in fruit aroma, and become more abundant after storage [28,29]. As shown in Table 2, total ester content increased from 32.93 to 36.29 mg/kg FW after 14 days storage at 4 °C, and then rapidly reached 60.18 and 63.43 mg/kg FW after a further 7 days storage at 4 °C and 20 °C, respectively. In general, esters with various carbons exhibited distinct patterns (Table 2). Butyl acetate (C6) and hexyl acetate (C8) were the crucial esters in pear at each stage, and their content experienced a significant increase. This finding was similar to that in a previous study, in which acetyl esters yielded a strong production in Bartlett pears during storage [30]. The contents of C9–C10 esters exhibited no significant difference between fruits at various storage stages (Table 2). The content of hexyl hexanoate (C12) decreased from 0.30 to 0.11 mg/kg FW during 21 days storage at 4 °C. However, C9–C12 esters had a 2–4-fold increase after 7 days at 20 °C. Thus, C9–C12 esters might be the specific aroma-active VOCs in Dr. Guyot pear after storage at room temperature [29]. During the storage period at 4 °C, the content of C17–C19 esters notably decreased from 0.14–2.12 mg/kg FW to undetectable. On the contrary, the content of ethyl oleate (C20) gradually increased during 21 days storage at 4 °C (from 0.14 to 0.40 mg/kg FW). Specially, its content decreased to 0.09 mg/kg FW after transfer to 20 °C. It is well known that the greatest amounts of flavor compounds are derived from esters during the growth of the fruit [31]. Therefore, C17–C20 esters might be the precursors of flavor compounds in pears after harvest.

As shown in Table 2, 12 alcohol compounds with content of at least 0.10 mg/kg FW were detected in Dr. Guyot pear at the beginning of the storage period. After 21 days storage at 4 °C, five of these compounds became undetectable. Specially, the total content of alcohols increased from 6.45 to 16.47 mg/kg FW after 14 days storage at 4 °C, and exhibited a slight decrease at the remaining stages. However, only five of the alcohols identified had strong production, especially 1,3-octanediol, the content of which increased from undetectable to 11.06 mg/kg FW. Storage at 20 °C contributed to an obvious degradation of 1,3-octanediol (from 11.06 to 5.28 mg/kg FW), which was the main reason for the decrease in total alcohol content (from 16.47 to 12.31 mg/kg FW) after 14 days.

As the primary contributors to fruit aroma, aldehydes are the predominant VOCs in immature fruit [27]. Nine of the aldehydes with content within the range of 0.10–0.88 mg/kg FW were provisionally identified in Dr. Guyot pear. During 14 days storage at 4 °C, the concentration of aldehydes showed a significant decrease, with four of them becoming absent (Table 2). According to previous studies, degradation of aldehydes in fruits during storage was caused by catalyzation of alcohol dehydrogenase (ADH) [32,33]. The only exception was hexanal, the content of which was relatively stable at all stages.

There were 3 ketones detected in pear with a concentration of 0.32–1.05 mg/kg FW, including 1-hepten-3-one, sulcatone, and damascenone. As shown in Table 2, storage led to a decrease in 1-hepten-3-one content, while, sulcatone and damascenone had a relatively stable content at all the stages.

Total content of alkenes gradually increased from 8.28 to 25.04 mg/kg FW during storage at 4 °C. Only the content of β-farnesene exhibited a stable or decreasing trend. Production of (Z,E)-α-farnesene, (E,E)-α-farnesene, and α-terpinene experienced a rapid rise during storage, increasing from 0.18-7.69 to 1.83-17.64 mg/kg FW. Storage at 20 °C result in a strong production of all alkene compounds, especially (E,E)-α-farnesene, which increased from 13.10 to 30.63 mg/kg FW. According to a previous study, rapid production of (E,E)-α-farnesene was associated with the accumulation of its synthase (*pMdAFS1*) in the skin of apple [34]. However, products of (E,E)-α-farnesene oxidation could induce superficial scald disorder, which is an important disease in the fruit industry. Therefore, the comparative analysis of (E,E)-α-farnesene biosynthesis could help to identify resistant and susceptible cultivars.

### 3.4. Conversion Pathway of VOCs in Pear

Most VOCs in food are generated by chemical reactions between substrates [26]. In this study, a series of flavor compounds with similar structure or synthesis route were detected, such as 2-octen-1-ol, octanol, octyl acetate, 2-octenal, 2-hexenyl acetate, hexyl acetate, 2-hexenal, hexanal, and hexanol. Those compounds could be formed from hydrogenation or dehydrogenation of other compounds.

According to Lara et al. (2003), volatile esters are generated by esterification of alcohols, and the reaction is catalyzed by the enzyme alcohol o-acyltransferase (AAT) [35]. As shown in Table 2, an increased content of octyl acetate (from 0.40 to 0.53 mg/kg FW) was associated with a decrease in 2-octen-1-ol (from 0.59 to undetectable) and 1-octanol (from 0.43 to 0.19 mg/kg FW) after 14 days storage. Furthermore, Dr. Guyot pear presented a reduced 2-octenal production after storage at 4 °C (from 0.75 to 0.31 mg/kg FW). Volatile alcohols are formed during the catalytic reactions of aldehydes and ADH [35]. Based on the results from the present study, it is possible that alcohols were the intermediates for conversion of aldehydes to esters (Figure S4a). However, this assumption was not confirmed due to the limitation of the experimental method used.

Similarly, pear fruit showed a rapid increase of hexyl acetate content (from 19.91 to 45.97 mg/kg FW) during storage. Meanwhile, 2-hexenal exhibited a decreasing trend (from 0.23 to 0.15 mg/kg FW). This result suggests that hexyl acetate was the 2-hexenal derived ester, and 1-hexanol was the intermediate (Figure S4b). Probably due to the intense ADH activity [32], 1-hexanol showed a slight increase during storage.

## 4. Conclusions

In this study, VOCs in pear fruit were analyzed using HS-SPME combined with GC-MS. Salt addition used during homogenization enhanced dissociation of VOCs and prevented browning of pear. A total of 121 VOCs were quantified in 12 different cultivars of pear fruit. The number and contents of VOCs in different pear cultivars varied dramatically, ranging from 13 to 71 and from 3.63 to 55.65 mg/kg FW, respectively. The Dr. Guyot cultivar showed the highest level of VOCs. The 12 pear cultivars could be divided into three groups on the basis of the results of PCA. The first group included one cultivar, Dr. Guyot. The second group consisted of three sand pear cultivars. The third group was composed of the other eight cultivars.

After 21 days of storage at 4 °C, the total concentration of VOCs increased from an initial content of 50.76, to 101.33 mg/kg FW. Storage at 20 °C largely contributed to a higher production of VOCs than storage at 4 °C. During 21 days of storage at 4 °C, the content of esters showed a gradual increase, while the content of alcohols and aldehydes decreased. Based on the results presented, the related alcohols were recognized as the intermediates in the conversion of aldehydes to esters in pear fruit after harvest.

## Figures and Tables

**Table 1:** The origin, group, scientific name, province, geographic point, sampling time, and characteristic taste of pear fruit of 12 cultivars.

Cultivars	Dr. Guyot	Starkrimson	Caiyunhong	Mantianhong	Meirensu	Yunhexue	Nanguo	Hanhong	Qiuyue	Yali	Yuluxiang	Zaosu
Origin	Europe	Europe	New Zealand	China	China	China	China	China	China	China	China	China
Group	Occidental pear	Occidental pear	Sand pear	Sand pear	Sand pear	A cross	Akiko pear	White pear	White pear	White pear	White pear	White pear
Scientific name	*Pyruscommunis* Linn	*Pyruscommunis* Linn	*Pyrus pyrifolia* (Burm. f.) Nakai	*Pyrus pyrifolia* (Burm. f.) Nakai	*Pyrus pyrifolia* (Burm. f.) Nakai	*-*	*Pyrus ussuriensis* Maxim	*Pyrus bretschneideri* Rehd	*Pyrus bretschneideri* Rehd	*Pyrus bretschneideri* Rehd	*Pyrus bretschneideri* Rehd	*Pyrus bretschneideri* Rehd
Province	Liaoning	Liaoning	Yunnan	Yunnan	Yunnan	Zhejiang	Liaoning	Jilin	Hebei	Hebei	Shanxi	Liaoning
Geographic point	41°48′ N, 123°25′ E	41°48′ N, 123°25′ E	25°22′ N, 102°25′ E	25°22′ N, 102°25′ E	25°22′ N, 102°25′ E	29°30′ N, 119°25′ E	41°48′ N, 123°25′ E	44°10′ N, 125°18′ E	38°03′ N, 114°26′ E	38°03′ N, 114°26′ E	38°10′ N, 112°09′ E	41°48′ N, 123°25′ E
Time (month/year)	08/2021	08/2021	08/2021	08/2021	08/2021	09/2021	10/2021	09/2021	09/2021	09/2021	09/2021	09/2021
Taste	Soft, Sweet	Soft, Sweet	Crisp, Sweet	Crisp, Sour, Sweet	Crisp, Sour, Sweet	Crisp, Sweet	Soft, Sour, Sweet	Crisp, Sweet	Crisp, Sweet	Crisp, Sweet	Crisp, Sweet	Crisp, Sweet

Note: Yunhexue is a cross between a Sand and a White pear.

**Table 2:** Contents of VOCs in Dr. Guyot pears during storage at 4 °C.

Compounds Name	Molecular Formula	Content of VOCs in Pears After Storage (mg/kg FW)			
		Before	14 Days	21 Days	14 Days + 7 Days at 20 °C
Butyl acetate	C_6_H_12_O_2_	7.18 ± 0.62	6.16 ± 0.51	11.42 ± 1.14	10.17 ± 1.21
Pentyl acetate	C_7_H_14_O_2_	0.40 ± 0.05	0.20 ± 0.03	0.48 ± 0.06	0.33 ± 0.03
Hexyl acetate	C_8_H_16_O_2_	19.91 ± 1.19	28.36 ± 2.56	45.97 ± 4.20	49.54 ± 4.6
Heptyl acetate	C_9_H_18_O_2_	0.16 ± 0.01	0.13 ± 0.02	0.16 ± 0.03	0.37 ± 0.05
Butyl hexanoate	C_10_H_20_O_2_	0.26 ± 0.03	0.05 ± 0.01	0.06 ± 0.01	0.11 ± 0.01
Hexyl butyrate	C_10_H_20_O_2_	0.20 ± 0.03	0.06 ± 0.01	0.11 ± 0.01	0.23 ± 0.02
Octyl acetate	C_10_H_20_O_2_	0.40 ± 0.03	0.53 ± 0.04	0.48 ± 0.04	2.03 ± 0.20
Hexyl hexanoate	C_12_H_24_O_2_	0.30 ± 0.04	0.10 ± 0.01	0.11 ± 0.01	0.30 ± 0.03
Methyl palmitate	C_17_H_34_O_2_	1.54 ± 0.16	0.14 ± 0.01	0.22 ± 0.02	0.09 ± 0.01
7-Hexadecenoic acid methyl ester	C_17_H_32_O_2_	0.18 ± 0.02	-	-	-
Ethyl palmitate	C_18_H_36_O_2_	0.14 ± 0.02	-	-	-
Methyl elaidate	C_19_H_36_O_2_	2.12 ± 0.26	0.34 ± 0.03	0.77 ± 0.06	0.17 ± 0.03
Ethyl oleate	C_20_H_38_O_2_	0.14 ± 0.01	0.22 ± 0.02	0.40 ± 0.05	0.09 ± 0.01
Subtotal		32.93 ± 3.58	36.29 ± 3.94	60.18 ± 6.21	63.43 ± 6.82
1-Butanol	C_4_H_10_O	0.84 ± 0.07	1.12 ± 0.14	1.17 ± 0.17	0.78 ± 0.09
1-Hexanol	C_6_H_14_O	1.14 ± 0.15	1.28 ± 0.12	1.91 ± 0.45	1.17 ± 0.19
1-Octen-3-ol	C_8_H_16_O	0.84 ± 0.06	-	-	-
3-Cyclopentyl-1-propanol	C_8_H_16_O	0.30 ± 0.04	0.41 ± 0.03	0.40 ± 0.05	1.09 ± 0.15
Linalool	C_10_H_18_O	0.12 ± 0.01	-	-	-
1-Octanol	C_8_H_18_O	0.43 ± 0.03	0.19 ± 0.03	0.13 ± 0.01	0.30 ± 0.04
2-Octen-1-ol	C_8_H_16_O	0.59 ± 0.07	-	-	-
1-Nonanol	C_9_H_20_O	0.13 ± 0.01	-	-	-
Z-4-Dodecenol	C_12_H_24_O	0.11 ± 0.01	-	-	-
Farnesol	C_15_H_26_O	0.22 ± 0.02	0.13 ± 0.01	0.16 ± 0.02	0.31 ± 0.02
Bergamotol	C_15_H_24_O	1.45 ± 0.16	0.99 ± 0.14	0.78 ± 0.07	1.67 ± 0.11
2-Decen-1-ol	C_10_H_20_O	0.09 ± 0.01	0.79 ± 0.08	0.82 ± 0.09	1.05 ± 0.16
1,3-Octanediol	C_8_H_18_O_2_	-	11.06 ± 1.23	9.42 ± 0.87	5.28 ± 0.64
3,5-Di-tert-butylphenol	C_14_H_22_O	0.19 ± 0.03	0.50 ± 0.06	0.66 ± 0.08	0.66 ± 0.09
Subtotal		6.45 ± 0.61	16.47 ± 1.73	15.45 ± 1.15	12.31 ± 1.31
1-Hepten-3-one	C_7_H_12_O	0.32 ± 0.04	0.16 ± 0.02	0.17 ± 0.02	0.17 ± 0.03
Sulcatone	C_8_H_14_O	0.36 ± 0.05	0.31 ± 0.03	0.42 ± 0.03	0.36 ± 0.05
Damascenone	C_13_H_18_O	1.05 ± 0.10	1.16 ± 0.11	1.05 ± 0.10	0.79 ± 0.06
Subtotal		1.73 ± 0.12	1.63 ± 0.12	1.64 ± 0.17	1.31 ± 0.15
Hexanal	C_6_H_12_O	0.12 ± 0.01	0.10 ± 0.01	0.14 ± 0.01	0.13 ± 0.01
2-Hexenal	C_6_H_10_O	0.23 ± 0.02	0.11 ± 0.01	0.15 ± 0.02	0.09 ± 0.02
2-Octenal	C_8_H_14_O	0.75 ± 0.06	0.31 ± 0.05	0.24 ± 0.03	0.30 ± 0.04
2-Nonenal	C_9_H_16_O	0.17 ± 0.03	0.09 ± 0.01	0.08 ± 0.01	0.13 ± 0.01
2-Decenal	C_10_H_18_O	0.88 ± 0.10	-	-	-
2,4-Nonadienal	C_9_H_14_O	0.17 ± 0.02	-	-	-
Geranial	C_10_H_16_O	0.10 ± 0.01	0.03 ± 0.00	0.05 ± 0.01	0.06 ± 0.01
2-Dodecenal	C_12_H_22_O	0.56 ± 0.07	-	-	-
2,4-Decadienal	C_10_H_16_O	0.12 ± 0.01	-	-	-
Subtotal		3.10 ± 0.29	0.64 ± 0.05	0.66 ± 0.09	0.71 ± 0.09
β-Farnesene	C_15_H_24_	0.23 ± 0.02	0.24 ± 0.02	0.13 ± 0.02	1.13 ± 0.15
(Z,E)-α-Farnesene	C_15_H_24_	0.18 ± 0.02	3.48 ± 0.34	5.44 ± 0.59	7.72 ± 0.71
(E,E)-α-Farnesene	C_15_H_24_	7.69 ± 0.71	13.10 ± 1.42	17.64 ± 1.62	30.63 ± 3.15
α-Terpinene	C_10_H_16_	0.18 ± 0.02	1.16 ± 0.13	1.83 ± 0.17	2.03 ± 0.25
Subtotal		8.28 ± 0.75	17.98 ± 1.82	25.04 ± 2.65	41.51 ± 4.26
Total		50.76 ± 5.52	71.38 ± 7.41	101.33 ± 11.14	117.96 ± 12.21

Note: FW means fresh weight; - means not detected; *n* = 3, equivalent of cyclohexanone.

## Data Availability

Data is contained within the article.

## Supplementary Materials

The following supporting information can be downloaded at: https://www.mdpi.com/article/10.3390/foods11233778/s1, Figure S1: Photographs of 12 pear cultivars fruits; Figure S2: Photographs of pear sample with NaCl addition before (a) and after (b) homogenization; Figure S3: Principal component analysis (PCA) of VOCs in 12 pear cultivars; Figure S4: The conversion pathway of VOCs in pear fruit during storage; Table S1: Concentration of VOCs in fruits of 12 pear cultivars.
