# Supplementary material for: Monitoring Volatile Organic Compounds in Different Pear Cultivars during Storage Using HS-SPME with GC-MS
Source: Foods. 2022 Nov 23;11(23):3778. doi: 10.3390/foods11233778 (PMC9735802; doi:10.3390/foods11233778)
Supplement: Supplementary file 1 [file foods-11-03778-s001.zip › foods-2016544-supplementary.pdf]

Supplementary Material

Monitoring Volatile Organic Compounds in Different Pear Cultivars During Storage Using HS-SPME With GC-MS

Guanwei Gao [1, 2], Xinnan Zhang [1, 3], Zhen Yan [1, 2], Yang Cheng [1, 2], Haifei Li [1, 2], Guofeng Xu [1, 2]*

[1] Ministry of Agriculture and Rural Affairs, Institute of Pomology, Chinese Academy of Agricultural Sciences, Xingcheng 125100, China

[2] Laboratory of Quality & Safety Risk Assessment for Fruit, Xingcheng 125100, China

[3] Key Laboratory of Germplasm Resources Utilization of Horticultural Crops, Ministry of Agriculture and Rural Afffairs, Xingcheng 125100, China

* Correspondence: xuguofeng@caas.cn

Table of content

I. Photographs of 12 pear cultivars fruits.......... S3

II. Photographs of pear sample with NaCl addition before and after homogenization.......... S4

III. Concentration of VOCs in fruits of 12 pear cultivars.......... S5

IV. Principal component analysis (PCA) of VOCs in 12 pear cultivars.......... S11

V. The conversion pathway of VOCs in pear fruit during storage.......... S12

I. Photographs of 12 pear cultivars fruits

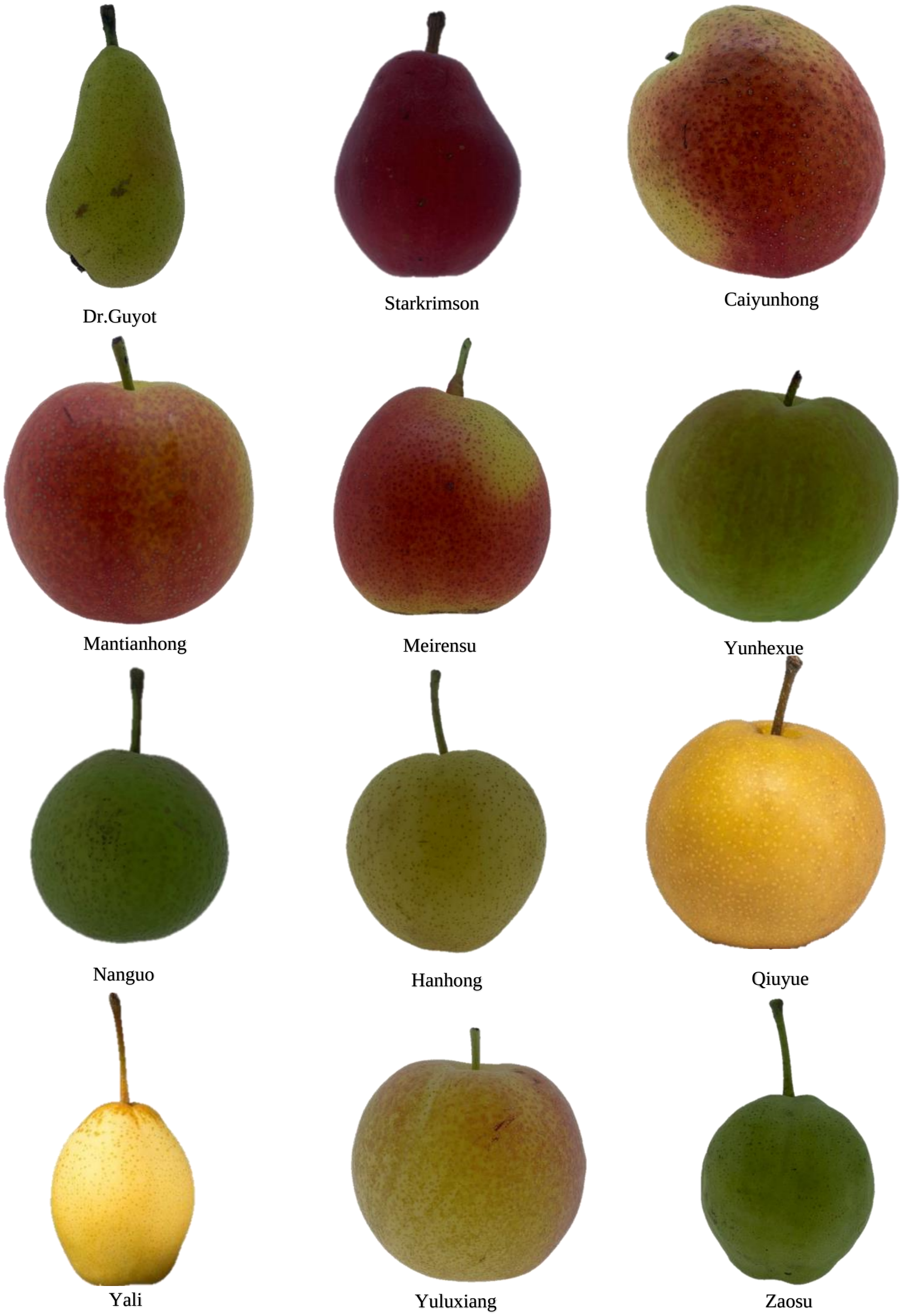

Figure S1. Photographs of 12 pear cultivars fruits.

II. Photographs of pear sample with NaCl addition before and after homogenization

Salt was widely used in previous studies to enhance the dissociation of VOCs. At first, 5.0 g of sliced pear fruit and 5.0 g NaCl were placed into a SPME vial. Browning of pear fruit occurred rapidly and resulted in color changes (Figure S2b) and loss of flavor. In this study, NaCl was directly mixed with sliced pear fruit (1:1, m/m) before homogenization. As shown in Figure S2a, the color of the pear matrix was less intense and prevented browning from developing.

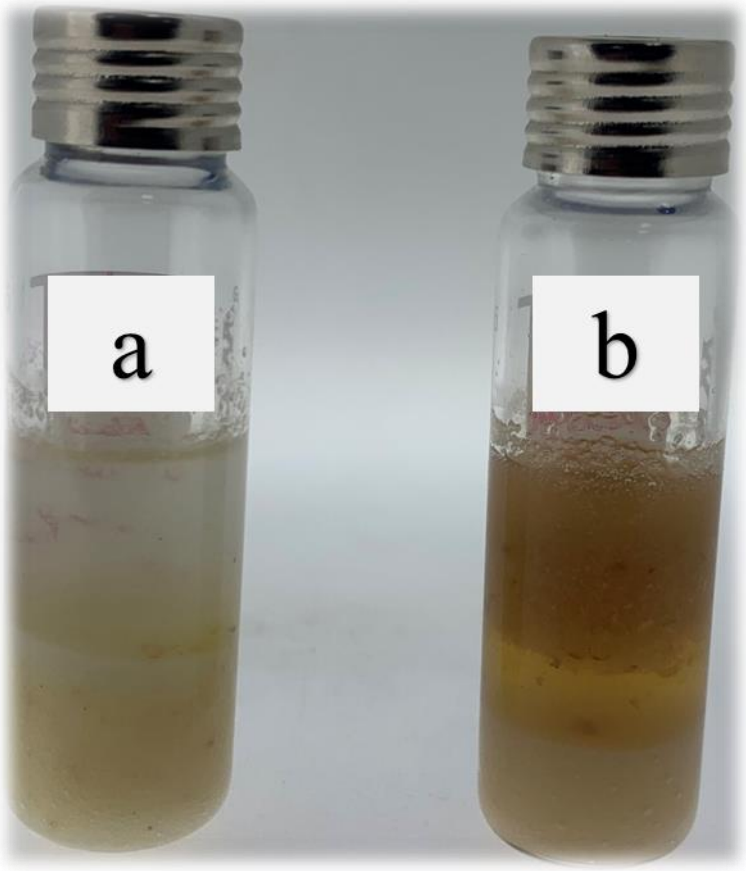

Figure S2. Photographs of pear sample with NaCl addition before (a) and after (b) homogenization.

III. Concentration of VOCs in fruits of 12 pear cultivars

Table S1. Concentration of VOCs in fruits of 12 pear cultivars.

	Compounds	RT (min)	Concentration Of VOCs (mg/kg FW)											
			Dr. Guyot	Starkrimson	Caiyunhong	Mantianhong	Meirensu	Yunhexue	Nanguo	Hanhong	Qiuyue	Yali	Yuluxiang	Zaosu
Ester														
1	Ethyl butyrate	10.62	-	-	1.07	1.06	0.31	-	-	0.02	-	-	-	-
2	Ethyl 2-methylbutyrate	11.19	-	-	0.12	0.12	-	-	-	-	-	-	-	-
3	Butyl acetate	11.97	7.18	0.23	-	-	-	0.09	0.06	0.01	0.01	-	-	0.01
4	Isoamyl acetate	14.26	-	-	-	0.02	-	-	-	-	-	-	-	-
5	Ethyl valerate	14.83	-	-	0.07	0.04	-	-	-	-	-	-	-	-
6	Ethyl 2-butenoate	16.50	-	-	0.09	0.13	-	-	-	-	-	-	-	-
7	Pentyl acetate	16.91	0.40	0.05	-	-	-	-	-	-	-	-	-	-
8	Butyl butyrate	19.45	0.03	-	-	-	-	-	-	-	-	-	-	-
9	Ethyl hexanoate	20.37	-	-	0.62	0.50	0.30	-	-	-	-	-	-	-
10	Ethyl tiglate	20.70	-	-	0.08	0.12	-	-	-	-	-	-	-	-
11	Hexyl acetate	22.78	19.91	0.63	0.21	-	0.05	0.11	0.12	0.09	-	0.03	0.06	0.15
12	Ethyl heptanoate	26.66	-	-	0.05	0.04	0.02	-	-	-	-	-	-	-
13	2-Hexenyl acetate	26.80	-	-	0.08	-	-	0.03	-	-	-	-	0.02	0.06
14	Ethyl 2-hexenoate	27.53	-	-	0.07	0.07	-	-	-	-	-	-	-	-
15	Heptyl acetate	29.31	0.16	-	-	-	-	-	-	-	-	-	-	-
16	Butyl hexanoate	31.83	0.26	0.04	-	-	-	-	-	-	-	-	-	-
17	Hexyl butyrate	32.02	0.20	0.03	-	-	-	-	-	-	-	-	-	0.04
18	Ethyl octanoate	33.27	-	-	0.18	1.33	1.65	-	-	-	-	-	-	-

19	Octyl acetate	35.95	0.40	-	-	0.02	0.20	-	-	-	-	-	-	-
20	2-Octenoic acid, ethyl ester	41.01	-	-	0.04	0.02	-	-	-	-	-	-	-	-
21	Hexyl hexanoate	44.44	0.30	0.04	-	-	-	-	-	-	-	-	-	0.17
22	Ethyl decanoate	46.21	-	-	-	0.07	0.04	-	-	-	-	-	-	-
23	Ethyl 4-decenoate	47.97	-	-	-	0.12	-	-	-	-	-	-	-	-
24	Ethyl benzoate	48.32	-	-	0.18	0.88	0.04	-	-	0.08	-	-	0.03	-
25	Ethyl 3-hydroxyhexanoate	48.95	-	-	0.04	0.05	-	-	-	-	-	-	-	-
26	Methyl salicylate	54.90	-	-	-	-	-	-	-	0.20	-	1.39	0.31	-
27	Ethyl phenylacetate	55.33	-	-	-	0.19	-	-	-	-	-	-	-	-
28	Phenethyl acetate	57.04	-	-	-	0.61	-	-	-	0.25	-	-	0.09	-
29	Ethyl (2E,4Z)-deca-2,4-dienoate	58.22	-	-	0.22	0.18	0.07	-	-	-	-	-	-	-
30	Methyl tetradecanoate	67.15	-	0.05	-	-	-	-	-	-	-	-	-	-
31	Methyl pentadecanoate	71.06	-	0.03	-	-	-	-	-	-	-	-	-	-
32	γ-Decalactone	72.35	-	0.02	0.15	0.05	0.07	0.05	0.03	-	-	0.04	0.04	-
33	Acetyleugenol	73.01	-	-	-	-	-	-	-	-	-	-	0.04	-
34	Methyl palmitate	73.94	1.54	1.48	0.07	0.02	-	0.02	0.06	0.09	0.03	0.01	0.03	0.20
35	7-Hexadecenoic Acid methyl ester	74.54	0.18	0.19	-	-	-	-	-	-	-	-	-	-
36	Ethyl palmitate	74.92	0.14	-	-	-	-	-	-	-	-	-	-	-
37	Methyl linolelaidate	75.86	0.04	-	-	-	-	-	-	-	-	-	-	-

38	Methyl stearate	79.70	0.20	0.07	-	-	-	-	-	-	-	-	-	-
39	Methyl elaidate	80.47	2.12	1.66	-	-	-	-	-	-	-	-	-	0.04
40	Ethyl oleate	81.60	0.14	-	-	-	-	-	-	-	-	-	-	-
	Subtotal		33.20	4.52	2.15	4.46	2.44	0.30	0.27	0.72	0.04	1.47	0.62	0.67
Alcohol														
1	1-Butanol	15.41	0.84	-	-	-	-	-	-	-	-	-	-	-
2	2-Methyl-1-butanol	18.76	0.09	0.03	-	-	-	-	-	-	-	-	-	-
3	1-Pentanol	21.33	-	0.04	-	-	-	-	-	-	-	-	-	-
4	Isoamyl alcohol	21.35	0.06	-	-	-	-	-	-	-	-	-	-	-
5	1-Hexen-3-ol	21.46	-	-	-	-	-	-	-	-	-	-	-	0.04
6	Acetic acid--hex-4-en-1-ol	25.71	-	0.06	-	-	-	-	-	0.09	-	-	-	0.05
7	1-Hexanol	27.91	1.14	0.53	0.15	0.29	0.05	1.33	0.27	0.21	1.62	1.56	1.24	3.12
8	3-Hexen-1-ol	30.06	-	0.14	-	-	-	-	0.22	0.10	0.13	0.19	0.04	0.06
9	2-Nonen-1-ol	30.66	-	-	-	-	-	0.17	-	-	-	-	-	-
10	Linalyl oxide	33.81	0.02	0.05	0.05	0.07	0.09	-	0.32	0.26	-	-	-	0.07
11	1-Octen-3-ol	34.30	0.84	0.50	0.04	-	-	0.04	0.23	-	-	-	-	-
12	1-Heptanol	34.57	0.04	0.10	-	-	-	-	-	-	-	-	-	-
13	2-Ethylhexanol	36.86	-	-	-	-	0.03	-	-	-	-	-	-	-
14	3-Cyclopentyl-1-propanol	39.37	0.30	-	-	-	-	-	-	-	-	-	-	-
15	Linalool	40.59	0.12	-	0.06	0.03	0.04	-	-	-	-	-	-	-
16	1-Octanol	41.17	0.43	0.21	0.13	0.18	0.27	0.06	0.10	0.18	-	0.03	0.04	0.04
17	2-Octen-1-ol	44.87	0.59	0.32	-	-	-	0.04	0.11	-	-	-	-	-
18	1-Nonanol	47.50	0.13	0.13	0.09	0.06	0.02	0.05	0.06	0.06	0.04	0.05	0.05	-
19	α-Terpineol	49.90	-	-	0.10	0.07	0.07	-	-	-	-	-	-	-

20	(E)-Dec-3-en-2-ol	50.10	-	-	-	0.08	0.02	-	-	-	-	-	-	-
21	1-Dodecanol	53.54	-	0.04	-	-	-	-	-	-	-	-	-	-
22	1-Decanol	53.57	-	-	-	0.11	0.06	-	-	-	-	0.02	-	-
23	Citronellol	53.85	0.08	-	-	-	-	-	-	-	-	-	-	-
24	Z-4-Dodecenol	55.58	0.11	-	0.23	-	0.32	-	-	-	-	-	-	-
25	5-Decen-1-ol	55.59	-	-	-	0.58	-	-	-	-	-	-	-	-
26	3,7-Dimethylocta-2,6-dien-1-ol	58.49	0.03	-	-	-	-	-	-	-	-	-	-	-
27	(2Z,6E)-Farnesol	59.80	0.22	-	-	-	-	-	-	-	-	-	-	-
28	Bergamotol	60.13	1.45	-	-	-	-	-	-	-	-	-	-	-
29	Butylated hydroxytoluene	62.24	-	-	-	-	-	0.02	-	-	-	-	-	-
30	2-Decen-1-ol	68.31	0.09	-	-	-	-	-	-	-	-	-	-	-
31	5-Hexen-1-ol	70.01	0.02	-	-	-	-	-	-	-	-	-	-	-
32	3,5-Di-tert-butylphenol	76.70	0.19	0.10	0.60	0.58	0.52	0.02	0.31	0.09	0.60	0.35	0.02	0.02
	Subtotal		6.79	2.25	1.45	2.05	1.49	1.73	1.62	0.99	2.39	2.20	1.39	3.40
Aldehyde														
1	Hexanal	12.44	0.12	0.10	0.36	0.25	0.19	0.33	0.28	0.44	0.44	0.48	2.00	0.84
2	2-Hexenal	19.70	0.23	0.32	0.83	0.64	0.21	0.76	0.80	1.19	0.11	0.29	4.60	2.88
3	Octanal	23.92	0.05	-	0.05	0.06	0.09	-	0.06	0.18	-	-	-	-
4	2-Heptenal	26.25	1.27	0.19	-	-	-	-	1.19	0.25	-	-	-	-
5	Nonanal	30.70	0.16	0.12	0.68	0.84	0.79	-	0.25	0.81	0.17	0.25	0.25	0.17
6	2-Octenal	33.11	0.75	0.21	0.20	0.03	0.05	0.05	0.89	0.36	0.03	0.03	0.04	0.03
7	Decanal	37.53	0.04	-	0.13	0.03	-	0.05	0.08	0.04	0.07	0.07	0.04	-
8	Benzaldehyde	39.51	-	0.04	0.20	-	-	-	-	-	-	0.03	-	-

9	2-Nonenal	39.94	0.17	-	0.10	-	0.05	-	0.07	0.06	-	-	-	-
10	2-Decenal	46.68	0.88	0.24	0.08	0.15	0.22	0.02	0.73	0.40	-	-	0.03	-
11	2,4-Nonadienal	50.28	0.17	-	-	-	-	-	-	0.06	-	-	-	-
12	Geranial	52.05	0.10	-	-	-	-	-	-	-	-	-	-	-
13	2-Undecenal	53.12	-	0.15	-	-	-	-	-	-	-	-	-	-
14	2-Dodecenal	53.13	0.56	-	0.04	-	-	-	0.33	0.07	-	-	-	-
15	(E,Z)-2,4-Decadienal	54.02	0.08	-	-	-	-	-	-	-	-	-	-	-
16	2,4-Decadienal	56.60	0.12	-	-	-	-	-	0.05	-	-	-	-	-
	Subtotal		4.70	1.37	2.67	2.00	1.60	1.21	4.73	3.86	0.82	1.15	6.96	3.92
Alkene														
1	Myrcene	16.23	0.05	-	-	-	0.03	-	-	-	-	-	0.04	-
2	(E)-β-Ocimene	20.40	0.03	-	-	-	-	-	-	-	-	0.02	0.04	-
3	Limonene	18.26	0.03	0.09	-	0.06	0.06	-	-	-	-	-	-	-
4	β-Ocimene	21.48	-	0.76	-	0.02	0.02	-	-	-	-	-	-	-
5	2,6-Dimethyl-2,6-octadiene	31.34	0.37	-	-	-	-	-	-	-	-	-	-	-
6	3,5,5-Trimethyl-2-hexene	36.67	0.03	-	-	-	-	-	-	-	-	-	-	0.22
7	β-Farnesene	47.91	0.23	0.13	-	-	-	-	-	0.06	-	-	-	0.19
8	Isomyocorene	50.57	-	-	-	-	-	-	-	-	-	-	0.03	-
9	9-Octadecene	50.79	-	-	-	-	-	-	-	-	-	-	-	-
10	(Z,E)-α-Farnesene	51.49	0.18	-	-	-	-	-	-	-	-	-	-	0.84
11	(E,E)-α-Farnesene	52.84	7.69	-	-	-	-	-	-	0.13	-	-	-	29.51
12	α-Terpinene	58.89	0.18	-	-	-	-	-	-	-	-	-	-	-
13	1-Hexacosene	76.47	-	-	-	0.07	-	-	-	-	-	-	-	-

	Subtotal		8.79	0.98	-	0.15	0.11	-	-	0.19	-	0.02	0.11	30.76
Ketone														
1	4-Hexen-3-one	11.82	0.03	0.02	-	-	-	-	0.03	0.01	-	-	-	-
2	3-Octanone	21.68	0.06	0.13	-	-	-	0.02	0.06	-	-	-	-	-
3	2-octanone	23.64	-	-	0.05	-	-	0.02	-	-	0.11	-	-	-
4	1-Hepten-3-one	24.72	0.32	0.09	0.14	-	-	0.02	0.34	0.18	-	-	-	-
5	Sulcatone	27.08	0.36	0.11	0.04	-	-	0.02	0.11	0.07	-	0.02	0.03	0.22
6	2-Undecanone	43.81	-	-	-	-	-	-	-	-	-	0.02	-	-
7	Damascenone	57.23	1.05	0.95	0.21	0.53	0.90	0.25	0.36	0.47	-	0.04	0.08	1.50
8	Geranylacetone	58.97	-	0.11	0.21	0.05	0.03	0.05	0.29	0.14	0.03	0.03	0.03	-
9	6-Methoxy-1-tetralone	64.94	0.09	-	-	-	-	-	-	-	-	-	-	-
10	Phytone	71.45	0.03	-	0.02	-	-	-	0.06	-	-	-	-	-
11	Pseudoionone	71.88	0.02	-	-	-	-	-	-	-	-	-	-	-
	Subtotal		1.96	1.41	0.67	0.58	0.93	0.38	1.25	0.87	0.14	0.11	0.14	1.72
Acid														
1	Hexanoic acid	59.79	-	-	-	-	-	-	-	0.07	-	-	-	-
2	Octanoic acid	70.25	0.02	-	0.19	-	0.12	-	-	0.04	-	-	-	-
3	Nonanoic acid	73.26	-	-	3.81	0.16	2.31	-	-	0.33	0.38	0.08	0.04	-
4	Decanoic acid	76.15	-	-	0.24	-	0.29	-	-	0.03	-	-	-	-
	Subtotal		0.02	-	4.24	0.16	2.72	-	-	0.40	0.38	0.08	0.04	-

Note: RT means retention time; FW means fresh weight; - means not detected.

IV. Principal component analysis (PCA) of VOCs in 12 pear cultivars

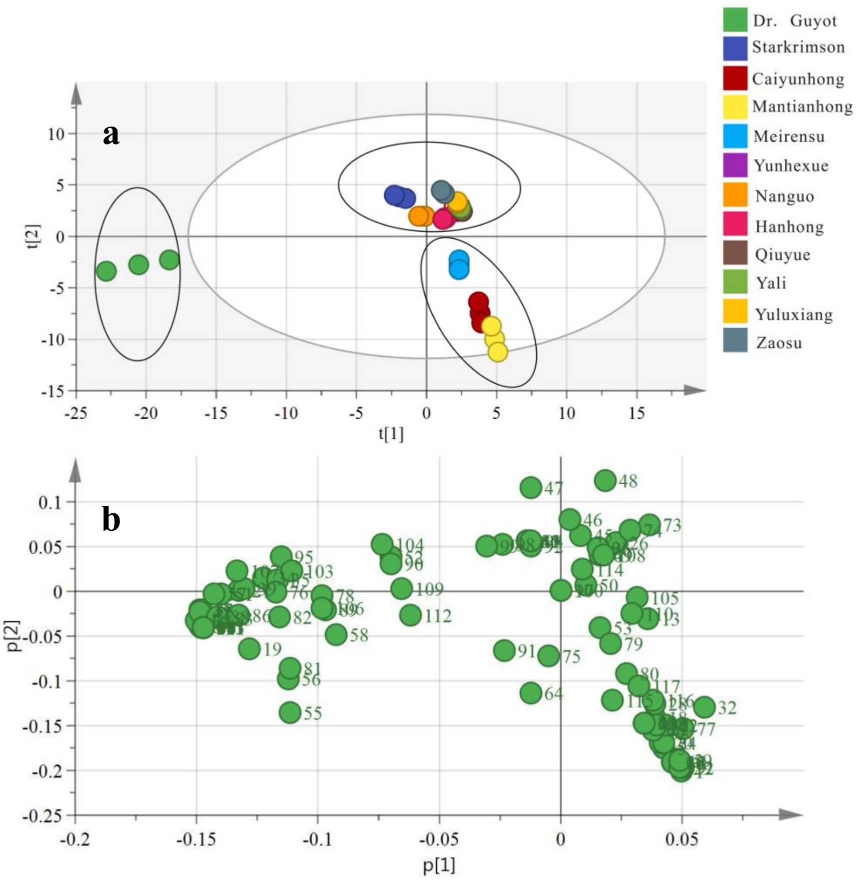

Figure S3. Principal component analysis (PCA) of VOCs in 12 pear cultivars. Note: (a) Scores scatter plot of PCA and (b) loadings plot of PCA analysis.

v. The conversion pathway of VOCs in pear fruit during storage

In this study, a series of flavor compounds with similar structure or synthesis route were detected, such as 2-octen-1-ol, octanol, octyl acetate, 2-octenal, 2-hexenyl acetate, hexyl acetate, 2-hexenal, hexanal, and hexanol. Those compounds could be formed from hydrogenation or dehydrogenation of other compounds.

As shown in Table 2, an increased content of octyl acetate (from 0.40 to 0.53 mg/kg FW) was associated with a decrease in 2-octen-1-ol (from 0.59 to undetectable) and 1-octanol (from 0.43 to 0.19 mg/kg FW) after 14 days storage. Furthermore, Dr. Guyot pear presented a reduced 2-octenal production after storage at 4 °C (from 0.75 to 0.31 mg/kg FW). Based on the results from the present study, it is possible that alcohols were the intermediates for conversion of aldehydes to esters (Figure S4a). However, this assumption was not confirmed due to the limitation of the experimental method used.

Similarly, pear fruit showed a rapid increase of hexyl acetate content (from 19.91 to 45.97 mg/kg FW) during storage. Meanwhile, 2-hexenal exhibited a decreasing trend (from 0.23 to 0.15 mg/kg FW). This result suggests that hexyl acetate was the 2-hexenal derived ester, and 1-hexanol was the intermediate (Figure S4b).

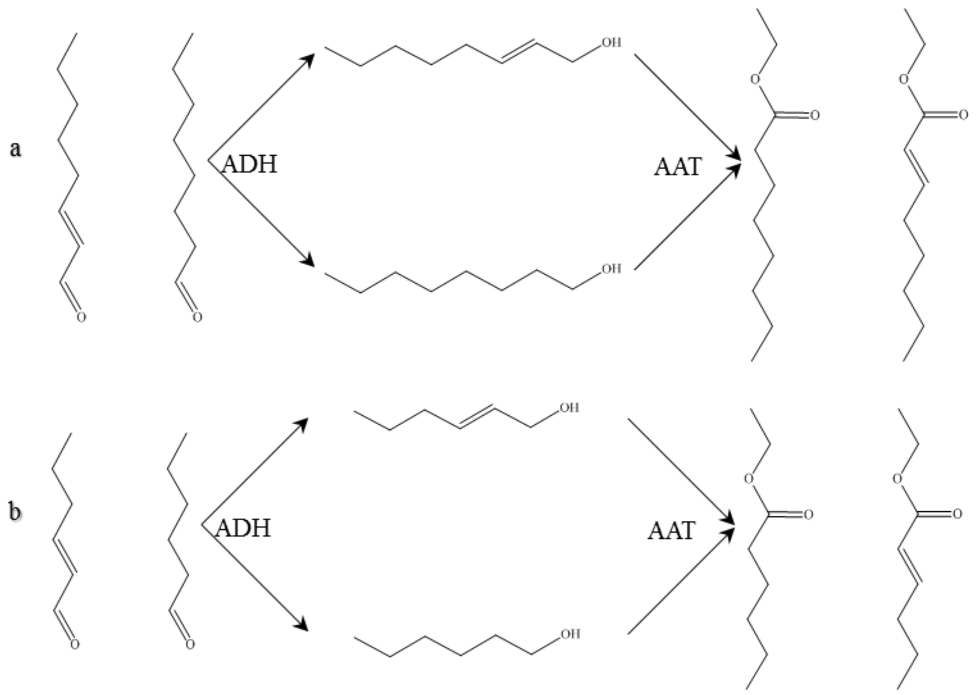

Figure S4. The conversion pathway of VOCs in pear fruit during storage. Note: ADH, alcohol dehydrogenase; AAT, alcohol o-acyltransferase.
